# Retargeting of UniCAR T cells with an *in vivo* synthesized target module directed against CD19 positive tumor cells

**DOI:** 10.18632/oncotarget.23556

**Published:** 2017-12-21

**Authors:** Dominik Bachmann, Roberta Aliperta, Ralf Bergmann, Anja Feldmann, Stefanie Koristka, Claudia Arndt, Simon Loff, Petra Welzel, Susann Albert, Alexandra Kegler, Armin Ehninger, Marc Cartellieri, Gerhard Ehninger, Martin Bornhäuser, Malte von Bonin, Carsten Werner, Jens Pietzsch, Jörg Steinbach, Michael Bachmann

**Affiliations:** ^1^ University Cancer Center, Carl Gustav Carus TU Dresden, Tumor Immunology, Dresden, Germany; ^2^ Helmholtz-Zentrum Dresden-Rossendorf, Institute of Radiopharmaceutical Cancer Research, Dresden, Germany; ^3^ GEMoaB Monoclonals GmbH, Dresden, Germany; ^4^ Leibniz Institute of Polymer Research Dresden, Dresden, Germany; ^5^ Cellex Patient Treatment GmbH, Dresden, Germany; ^6^ Medical Clinic and Policlinic I, University Hospital Carl Gustav Carus, TU Dresden, Dresden, Germany; ^7^ German Cancer Consortium, Carl Gustav Carus TU Dresden, Dresden, Germany; ^8^ National Center for Tumor Diseases, Dresden, Carl Gustav Carus TU Dresden, Dresden, Germany; ^9^ Department of Chemistry and Food Chemistry, School of Science, TU Dresden, Dresden, Germany

**Keywords:** CAR, CD19, retargeting, T cell, T cell therapy

## Abstract

Recent treatments of leukemias with T cells expressing chimeric antigen receptors (CARs) underline their impressive therapeutic potential but also their risk of severe side effects including cytokine release storms and tumor lysis syndrome. In case of cross-reactivities, CAR T cells may also attack healthy tissues. To overcome these limitations, we previously established a switchable CAR platform technology termed UniCAR. UniCARs are not directed against typical tumor-associated antigens (TAAs) but instead against a unique peptide epitope: Fusion of this peptide epitope to a recombinant antibody domain results in a target module (TM). TMs can cross-link UniCAR T cells with tumor cells and thereby lead to their destruction. So far, we constructed TMs with a short half-life. The fast turnover of such a TM allows to rapidly interrupt the treatment in case severe side effects occur. After elimination of most of the tumor cells, however, longer lasting TMs which have not to be applied via continous infusion would be more convenient for the patient. Here we describe and characterize a TM for retargeting UniCAR T cells to CD19 positive tumor cells. Moreover, we show that the TM can efficiently be produced *in vivo* from producer cells housed in a sponge-like biomimetic cryogel and, thereby, serving as an *in vivo* TM factory for an extended retargeting of UniCAR T cells to CD19 positive leukemic cells.

## INTRODUCTION

T cell-engaging bispecific antibodies (bsAbs, BiTEs) and T cells genetically modified to express chimeric antigen receptors (CARs) represent promising tools for immunotherapy of tumors [1–5]. Both BiTEs and CARs establish immune synapse-like interactions between T cells and cancer cells [6, 7]. Polarization of these complexes finally leads to activation of recruited CD8^+^ and CD4^+^ T cells and induces T cell-specific inflammatory and cytotoxic responses against the cross-linked target cells [8]. Until now, many groups including ours have established a series of bsAbs and CARs against potential tumor targets expressed on leukemic or solid cancer cells [9–23].

CARs currently used in the clinic consist of three domains: (i) an extracellular binding moiety, (ii) a transmembrane domain and (iii) an intracellular domain containing signaling motif(s). The extracellular binding domain is derived from a monoclonal antibody (mAb) by recombinant fusion of its variable heavy and light chain sequences. The transmembrane domain of CARs is most frequently derived from the CD28 or CD8 receptor [1–5, 24]. The intracellular signaling domains are taken from immune receptors. According to the number and origin of the signalling domains, CARs are devided into first, second and third generation CARs.

Currently, the most promising and mature clinical data for treating tumors with CAR T cells are available for the target antigen CD19 [24–27]. Unfortunately, CD19 is not exclusively expressed on malignant leukemic cells but also present on the surface of follicular dendritic cells and healthy B cells [28]. As a consequence, patients treated with CD19 CARs will develop a lifelong-lasting B cell aplasia. Nonetheless, this adverse effect does not prohibit the application of anti-CD19 CAR T cells as the lack of antibodies (Abs) can be compensated by intravenous immunoglobulin administration. One way to overcome long-lasting side effects associated with conventional CAR T cell therapy would be to eliminate the CAR T cells from succesfully treated patients using a suicide mechanism or an immunodepletion step [29–34].

**Figure 1:**
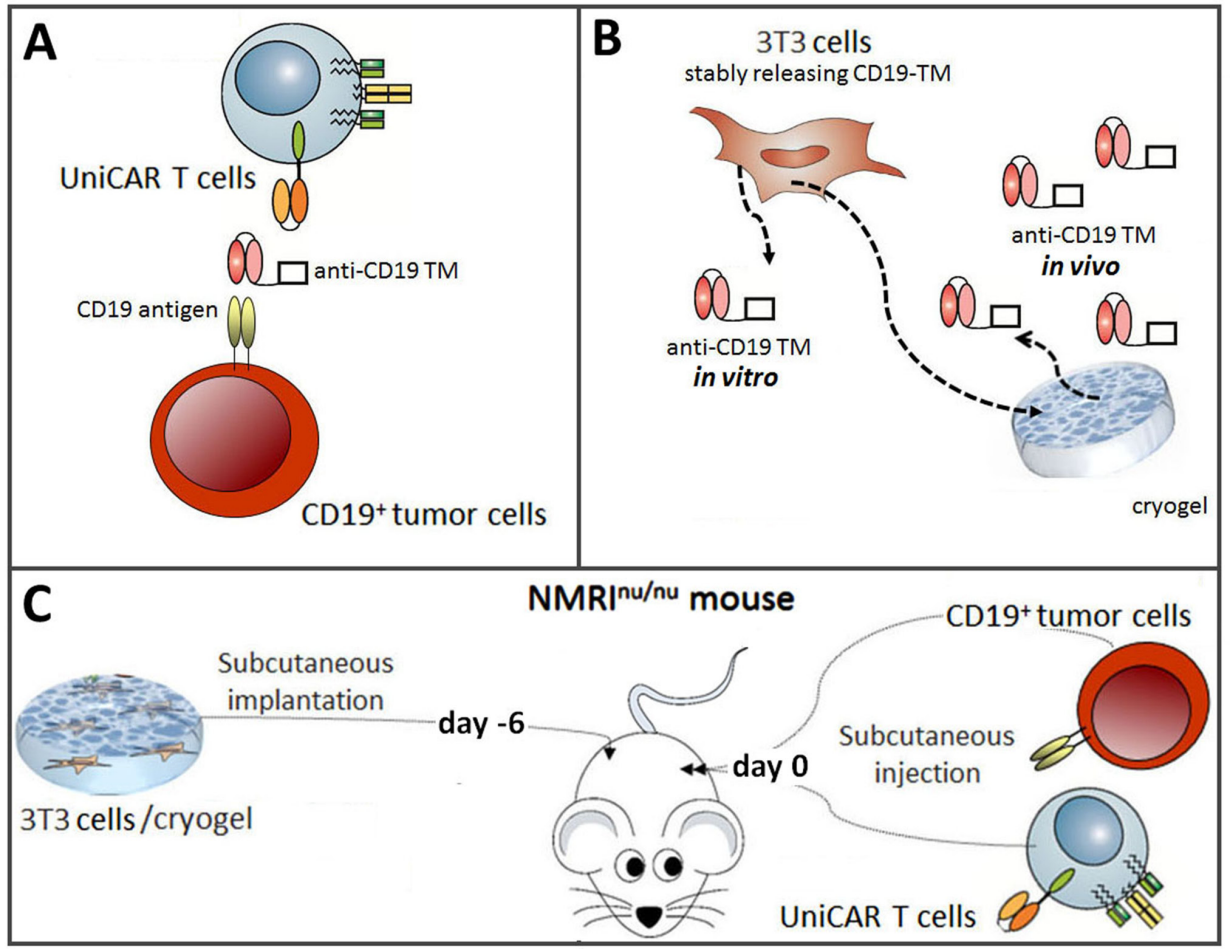
Schematic summary to show proof of concept for redirecting UniCAR T cells to tumor cells with an *in vivo* synthesized TM (**A**) Schematic view of the UniCAR system. For retargeting of UniCAR T cells to CD19 positive tumor cells a TM against the CD19 antigen (anti-CD19 TM) had to be constructed. In its presence, UniCAR T cells will be cross-linked to CD19 positive tumor cells which will finally lead to lysis of the latter. In the absence of the TM, UniCAR T cells will automatically be switched off. (**B**) For both *in vitro* and *in vivo* synthesis the reading frame encoding the anti-CD19 TM had to be transduced into a producer cell line. To this, murine 3T3 cells were selected. For *in vivo* synthesis the transduced cells were housed in starPEG-heparin cryogels. (**C**) For proof of concept, it had to be analyzed whether or not the amount of anti-CD19 TM that can be released form producer cells housed in the cryogel is sufficient for retargeting of UniCAR T cells to CD19 positive tumor cells *in vivo.*

If such a strategy also allows a rapid and complete shut down of CAR T cells in case of acute or even life-threatening immune reactions, however, has to be shown. Soon after antigen contact, the adoptively transferred CAR T cells will expand and produce inflammatory cytokines which can cause a clinically highly significant cytokine storm. In dependence on the tumor load, tumor lysis syndrome may also occur. Since the amount of tumor load can hardly be evaluated precisely at the time of adoptive transfer of CAR T cells neither their expansion nor related cytokine release or tumor lysis syndrome can be predicted. In case these side effects become life-threatening, the activity and function of the CAR T cells have to be stopped as fast as possible. In order to (i) avoid complete elimination of the genetically modified CAR T cells and (ii) realize a rapid and reversible off and on switch of CAR T cells, in 2014 we presented a modular CAR platform termed UniCAR [35]. UniCAR-equipped T cells are not directed to a cell surface target antigen and, therefore, are per se inert. They become active only in the presence of a TM which bridges UniCAR T cells and tumor cells (for a schematic view see Figure 1A). After elimination of the TM UniCAR T cells will automatically be turned off. Consequently, the activity of UniCAR T cells can be titrated and thereby potential side effects limited via infusion of the TM comparable to the application of BiTEs. A short elimination time of the TM is a prerequisite to rapidly shut down UniCAR T cells. TMs based on small recombinant Ab derivates such as single-chain fragment variables (scFvs) or nanobodies fullfill this criterium [36–41]. The downside of this safety mechanism is the permanently required infusion of the TM. Although favourable at the beginning of a UniCAR T cell therapy, a longer lasting TM that is not dependend on continous infusion would be more convenient for the patient when most of the tumor cells have been eradicated. Recently, we described the development of a cryogel-supported cell factory suitable for a sustained delivery of an anti-CD33-anti-CD3 bsAb, that is capable of specifically and efficiently redirecting CD3^+^ T lymphocytes towards CD33^+^ AML blasts [42, 43].

Here we describe (i) the development of an scFv-based TM against CD19 and (ii) its functionality for retargeting of UniCAR T cells *in vitro* and *in vivo*. Moreover, we show that cells transduced with the gene encoding the anti-CD19 TM can be housed in a macroporous four-arm poly(ethylene glycol) (starPEG)-heparin cryogel and used for *in vivo* production of the therapeutic molecule.

## RESULTS

The aims of the presented manuscript are schematically summarized in Figure 1: We wanted to (i) develop and functionally characterize a TM for redirection of UniCAR T cells to CD19 positive tumor cells (Figure 1A) and (ii) challenge the idea to manufacture the TM *in vivo* from the producer cell line housed in a starPEG-heparin cryogel (Figure 1B) for retargeting of UniCAR T cells in experimental mice (Figure 1C). For this purpose, we had to (i) clone the TM, (ii) establish a cell line permanently expressing the TM, (iii) isolate the TM from the supernatant, (iv) characterize the TM biochemically, (v) show its functionality *in vitro*, and finally (vi) demonstrate that the same producer cells when housed in the cryogel can release the TM into the blood stream at a sufficient concentration for treating experimental mice.

### Construction of the TM directed against CD19

**Figure 2:**
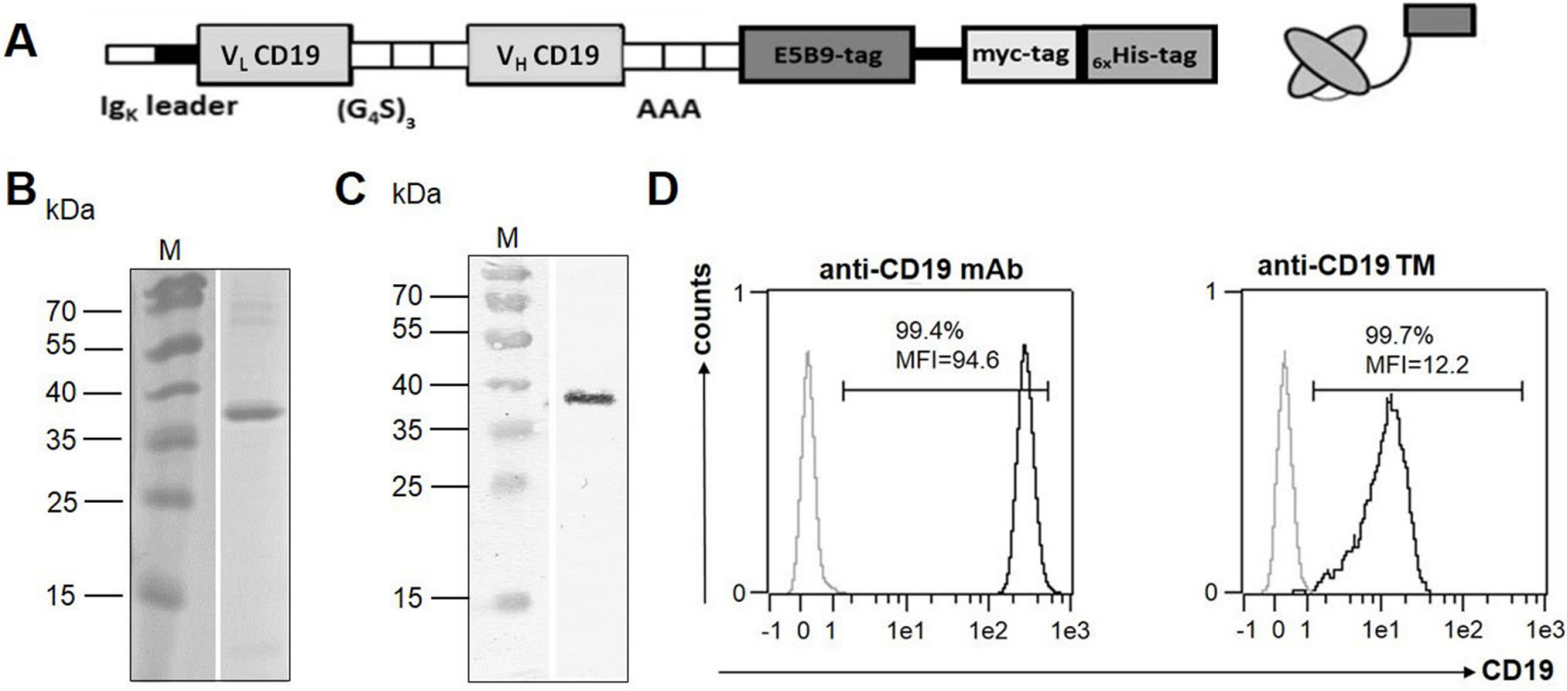
Construction, isolation and characterization of the anti-CD19 TM (**A**) Schematic structure of the murine anti-CD19 TM. (**B**) After expression, the anti-CD19 TM was isolated from cell culture supernatant by nickel-affinity chromatography. The elution fraction containing the purified anti-CD19 TM was separated via SDS-PAGE and subsequently stained with Coomassie brilliant blue G-250 or (**C**) transferred onto a nitrocellulose membrane and detected via its C-terminal His-tag by using an anti-his mAb. M, molecular weight marker. (**D**) Naturally CD19-expressing Nalm-6 cells were stained either with a commercially available anti-CD19/PE mAb (left) or with 20 µg/ml of the anti-CD19 TM (right) followed by incubation with the anti-UniCAR tag mAb and a PE-conjugated anti-mouse IgG secondary Ab. Finally, Nalm-6 cells were analyzed using flow cytometry. Cells stained with the corresponding isotype control mAb or the secondary Ab alone served as negative controls, respectively (grey graphs). Mean fluorescence intensity (MFI) and the percentage of positive cells are shown.

The TM against CD19 was constructed in an scFv format by fusing the sequences of the variable heavy and light chains of a previously described anti-CD19 mAb [44] with the UniCAR epitope sequence [39]. A schematic view of the anti-CD19 TM is shown in Figure 2A.

### Expression and isolation of anti-CD19 TM

For expression, the reading frame encoding the anti-CD19 TM was transduced into murine 3T3 cells and a permanent cell line expressing the anti-CD19 TM was established as described previously [45] [see also MATERIALS AND METHODS]. The anti-CD19 TM was expressed and purified from cell culture supernatants of the eukaryotic cells using nickel affinity chromatography [8] [see also MATERIALS AND METHODS].

A sample of the affinity-purified anti-CD19 TM was separated by SDS-PAGE (Figure 2B, 2C). After SDS-PAGE and staining of the gel with Coomassie Brilliant Blue G250 a prominent protein with a molecular weight of 37 kDa was visualized (Figure 2B) which reacted with the mAb against the UniCAR epitope [9] [see also MATERIALS AND METHODS] after immunoblotting (Figure 2C).

### Binding of the anti-CD19 TM to CD19 positive tumor cells

In order to verify the binding capability of the anti-CD19 TM naturally CD19-expressing cell lines were tested. Data shown in Figure 2D were obtained for CD19 positive Nalm-6 cells. Similar data were obtained for other CD19 positive cell lines including Daudi and Raji cells (data not shown). For analysis, 3 × 10^5^ Nalm-6 cells were stained with either a commercially available anti-CD19/PE mAb (Figure 2D, left panel) or 20 µg/ml of the anti-CD19 TM (Figure 2D, right panel) followed by staining with the mAb directed against the UniCAR epitope (anti-La5B9) and a PE-conjugated anti-mouse IgG secondary Ab. Stained cells were analyzed by flow cytometry [9] [see also MATERIALS AND METHODS]. According to these data (i) the constructed anti-CD19 TM is able to bind its target antigen CD19 and (ii) the UniCAR epitope is accessible in the native anti-CD19 TM.

In order to estimate the binding affinity, the *K*_D_ value was assessed as described previously (data not shown) [39]. Thereby, we determined a *K*_D_ value of 27 nM for the anti-CD19 TM.

### Activation of UniCAR T cells occurs in a TM-dependent- and target-specific manner

**Figure 3:**
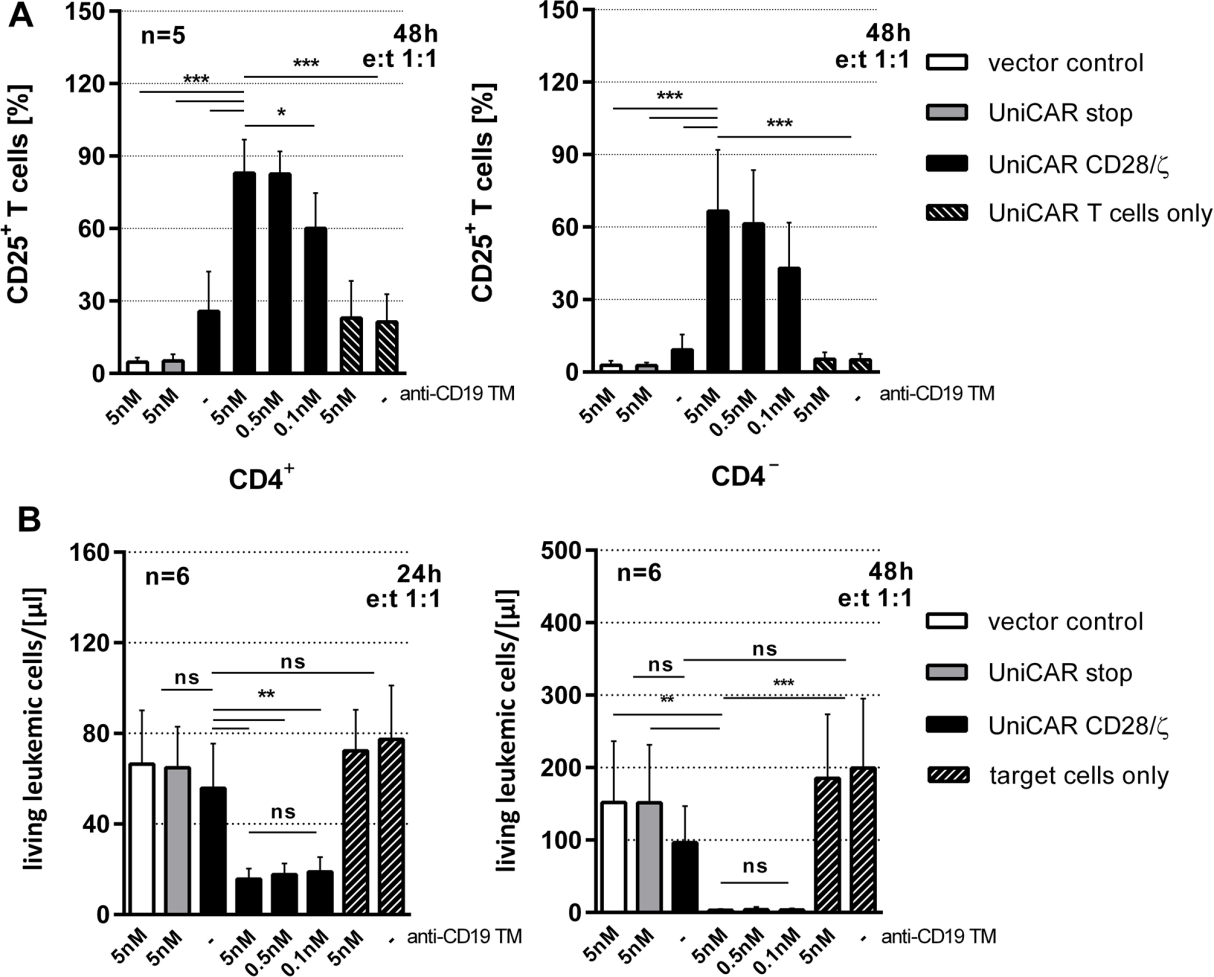
Retargeting of UniCAR T cells to CD19 positive target cells via the anti-CD19 TM eFluor670^®^-labeled Nalm-6 cells (1 × 10^4^) were incubated with T cells engrafted with either the vector control encoding the EGFP marker protein (vector control), the UniCAR stop construct lacking the intracellular signaling domain (UniCAR stop) or the UniCAR signaling construct (UniCAR CD28/ζ) at an e:t ratio of 1:1 in the presence or absence of 0.1 to 5 nM anti-CD19 TM. (**A**) After 48h activation of T cells was measured via surface expression of the activation marker CD25 on CD3-positive T cells for five individual T cell donors. CD3 cells were subdivided into CD4^+^ and CD4^-^ cells by fluorescence-staining with an anti-CD4 mAb. (**B**) After 24h or 48h of coculture numbers of surviving tumor cells were determined using a MACSQuant^®^ Analyzer and shown as living cells/µl (total assay volume 200 µl). Counts of surviving Nalm-6 cells and SD are reported for six different T cell donors. Statistical significance was assessed by one-way ANOVA with Bonferroni multiple comparison test (^*^p < 0.05, ^**^p < 0.01, ^***^p < 0.001; ns, not significant).

For analysis of activation, Nalm-6 cells (1 × 10^4^) were incubated with T cells engrafted with either the vector control encoding the EGFP marker protein (vector control), the UniCAR stop construct lacking the intracellular signaling domain (UniCAR stop) or the UniCAR signaling construct (UniCAR CD28/**ζ**) at an e:t ratio of 1:1 in the presence or absence of 0.1 to 5 nM anti-CD19 TM. After 48h of coculture activation of T cells was measured via surface expression of the activation marker CD25. As shown in Figure 3A, activation occurs for both CD4^+^ and CD8^+^ (measured as CD4^+^ and CD4^-^) UniCAR T cells in strict dependence of the anti-CD19 TM. T cells equipped with the vector control or UniCAR stop construct were not activated. Without target cells no UniCAR T cell stimulation occured neither in the presence nor absence of the anti-CD19 TM (Figure 3A).

### Killing of CD19 positive tumor cells by retargeted UniCAR T cells occurs in a TM-dependent- and target-specific manner

**Figure 4:**
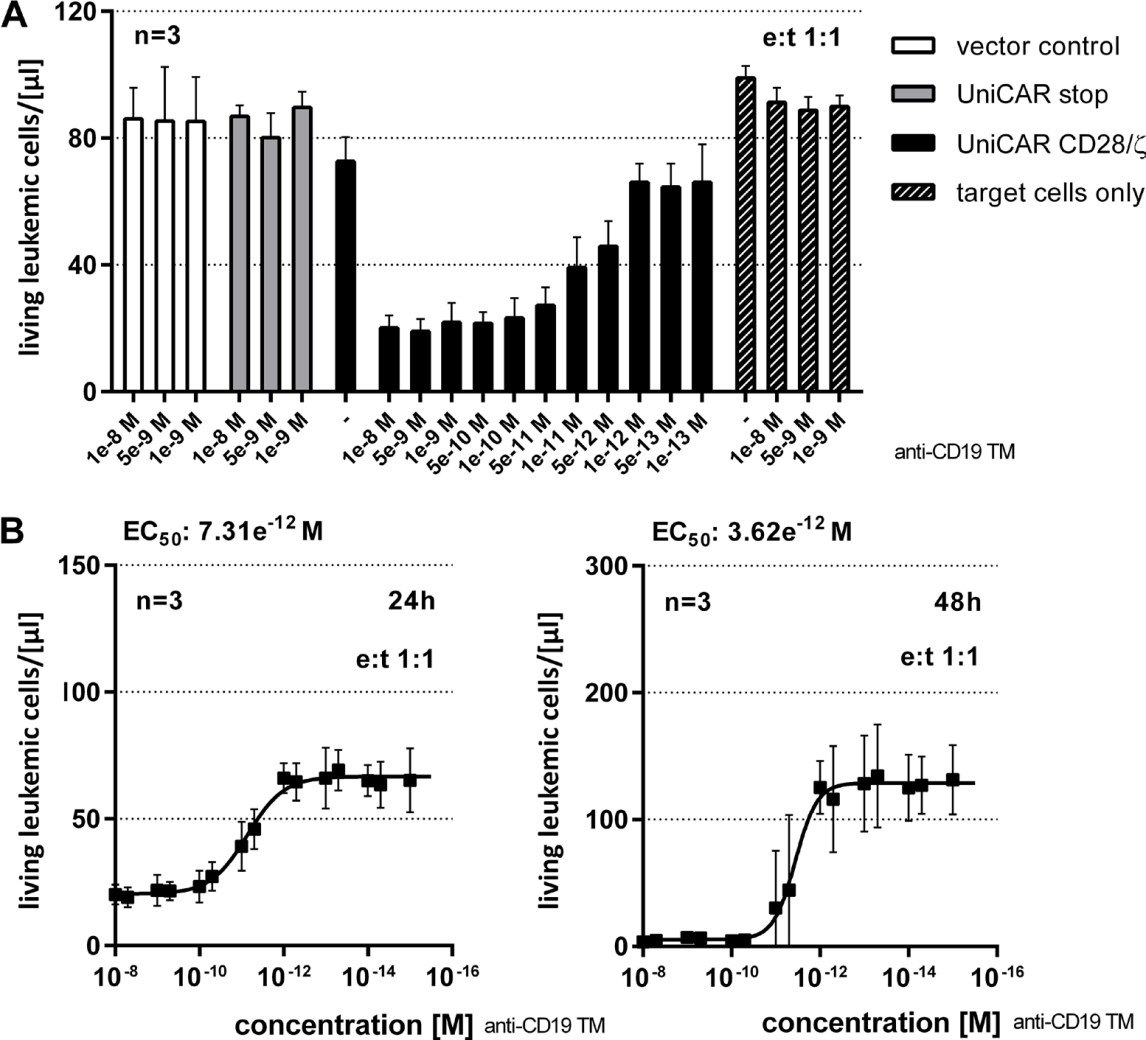
Estimation of EC_50_ value for the anti-CD19 TM (**A**) In order to estimate the range of working concentration for the anti-CD19 TM, 1x10^4^ eFluor670^®^-labeled Nalm-6 cells were cocultivated with human T cells modified with the vector control, the UniCAR stop or the UniCAR signaling construct at an e:t ratio of 1:1. The CD19-specific TM was added at indicated concentrations. Total cell numbers of surviving tumor cells were measured after 24 h using a MACSQuant^®^ Analyzer and shown as living cells/µl (total assay volume 200 µl). (**B**) EC_50_ values of the CD19-specific TM were determined for an e:t ratio of 1:1. After 24h and 48h of cultivation with UniCAR 28/ζ –armed T cells number of surviving tumor cells were determined using the MACSQuant^®^ Analyzer and shown as living cells/µl (total assay volume 200 µl).

For functional analysis, we used a FACS-based killing assay [38] [see also MATERIALS AND METHODS]. A total of 1 × 10^4^ Nalm-6 cells were labeled with eFluor670^®^ and incubated with T cells engrafted with the UniCAR signaling construct (Figure 3B, UniCAR CD28/ζ) at an e:t ratio of 1:1. T cells expressing either the vector control encoding EGFP marker protein (Figure 3B, vector control) or the UniCAR stop construct lacking the intracellular signaling domain (Figure 3B, UniCAR stop) served as negative controls. The number of surviving tumor cells was determined via flow cytometry after coculturing genetically modified T cells with CD19 positive tumor cells for 24h and 48h as indicated in the presence or absence of 0.1 nM to 5 nM of anti-CD19 TM. As shown in Figure 3B, only T cells equipped with a signaling UniCAR construct efficiently eliminate target cells. CD19 negative cells were not attacked by UniCAR T cells either in the presence or absence of the anti-CD19 TM (data not shown). Similar data were obtained for other CD19 positive tumor cells e.g. Raji and Daudi cells (data not shown). In order to estimate the EC_50_ value of the anti-CD19 TM, titration experiments were performed as described previously [39]. As shown in Figure 4, we estimated EC_50_ values of 7.3 pM after 24h and 3.6 pM after 48h, respectively. Our data show that lysis of CD19 positive tumor cells via the combination of the anti-CD19 TM and UniCAR T cells occurs in a TM-dependent- and TM-specific manner.

According to previous studies [36, 37, 40] a UniCAR TM cannot only be used in combination with UniCARs as effector system but also in combination with a universal bispecific effector module (EM) (For a schematic view see Supplemental Figure 1A). This EM is a bispecific Ab which on the one hand is directed to CD3 and on the other hand to the UniCAR epitope. Indeed, the anti-CD19 TM combined with this EM is capable of lysing CD19 positive tumor cells (Supplemental Figure 1B) including from patients with mixed lineage leukemia (MLL) or acute lymphoblastic leukemia (ALL) both in an hetero- and autologous setting (Supplemental Figure 1C).

### Release of cytokines by retargeted UniCAR T cells occurs in a TM-dependent- and target-specific manner

**Figure 5:**
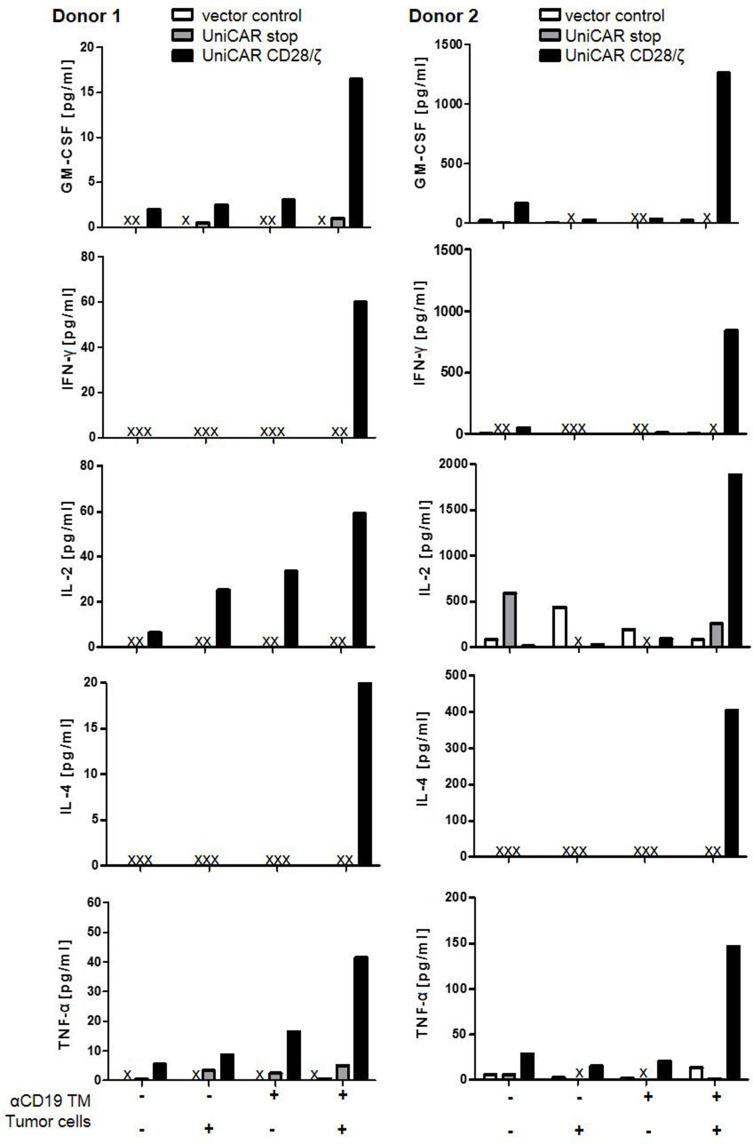
Estimation of released cytokines from CD19-retargeted UniCAR T cells Genetically engineered T cells were cultivated with or without tumor cells at an e:t ratio of 5:1, either in the presence or in the absence of 30 nM anti-CD19 TM for 24h. Released cytokines were measured using the MACSPlex Cytokine 12 Kit, human. Only the depicted cytokines GM-CSF, IFN-γ, IL-2, IL-4 and TNF-α could be detected under these conditions. Data are reported for two independent donors. X, not detectable.

For analysis of cytokine release, T cells genetically engineered to express either only EGFP (vector control), UniCAR stop or UniCAR CD28/ζ were cultivated with or without tumor cells at an e:t ratio of 5:1, either in the presence or absence of 30 nM of anti-CD19 TM for 24h. Released cytokines were estimated using the MACSPlex Cytokine 12 Kit, human [40] [see also MATERIALS AND METHODS]. This bead-based assay allowed us to detect and quantify the cytokines GM-CSF, IFN-α, IFN-γ, IL-2, IL-4, IL-5, IL-6, IL-9, IL-10, IL-12, IL-17A and TNF-α in parallel. Only the cytokines GM-CSF, IFN-γ, IL-2, IL-4 and TNF-α were detected at relevant concentrations in the presence of UniCAR CD28/ζ-armed T cells, tumor cells and TM substantiating that cytokine secretion occurs in a strictly TM- dependent- and target-specific manner (Figure 5).

### Specific killing of CD19 positive tumor cells by retargeted UniCAR T cells via anti-CD19 TMs released *in vivo* from cryogel-housed producer cells

**Figure 6:**
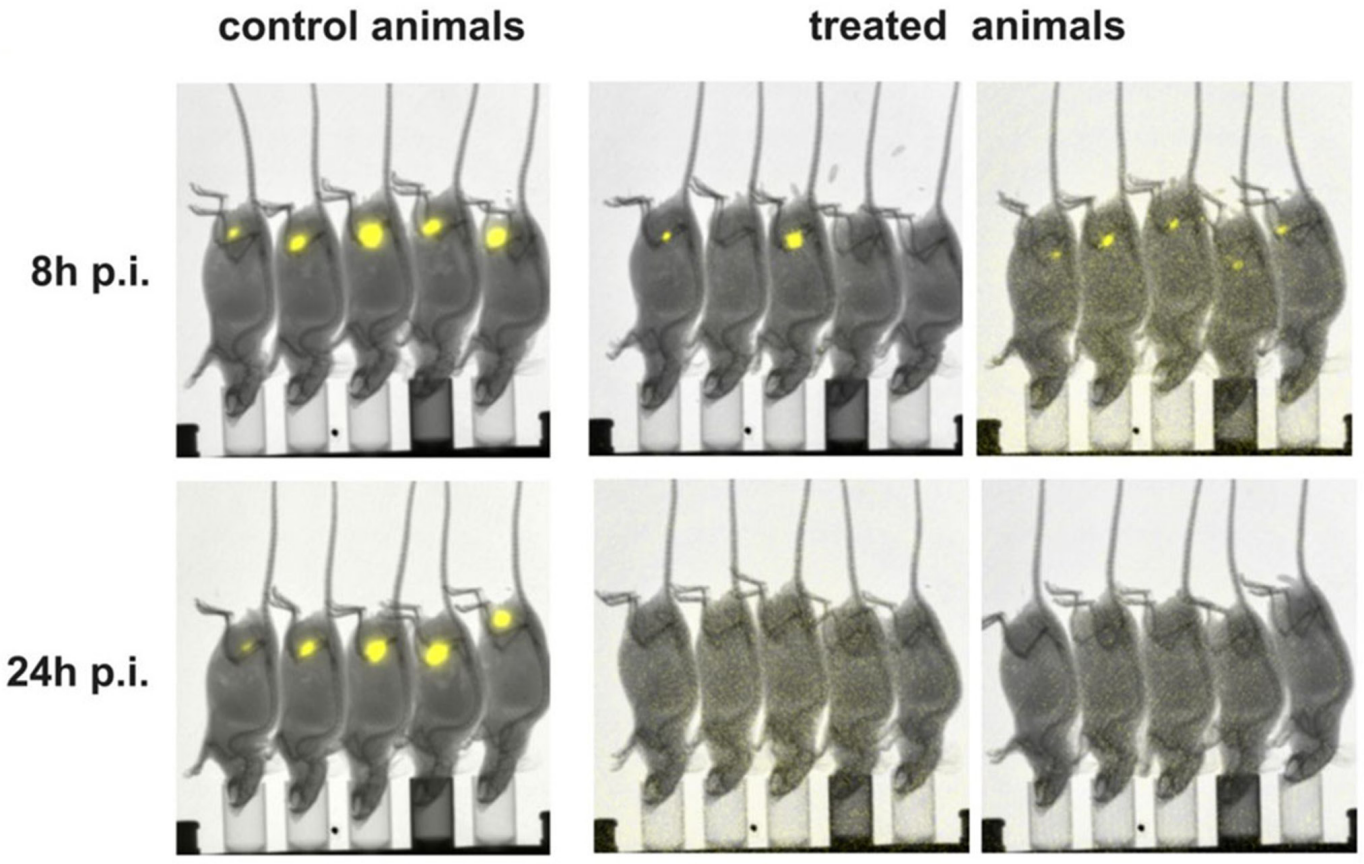
Retargeting of UniCAR T cells to CD19 positive tumor cells in experimental mice via *in vivo* synthesized anti-CD19 TM NMRI^nu/nu^ mice were s.c. transplanted with starPEG-heparin cryogels housing 5x10^5^ anti-CD19 TM-producing 3T3 cells (treated animals, *n* = 10) or empty scaffolds as a control (control animals, *n* = 5) into their left leg. Following six days post-transplantation, both animal groups were s.c. injected into their right legs with a mixture of 1 × 10^6^ UniCAR CD28/ζ-armed T cells and 1 × 10^6^ CD19 positive Nalm-6 cells expressing firefly luciferase. Luminescence imaging of anesthetized mice was performed 10 min after i.p. injection of 200 μl of D-luciferin potassium salt at 8h and 24h post-injection (p.i.). The acquired optical images of control animals and treated animals are reported, respectively.

**Figure 7:**
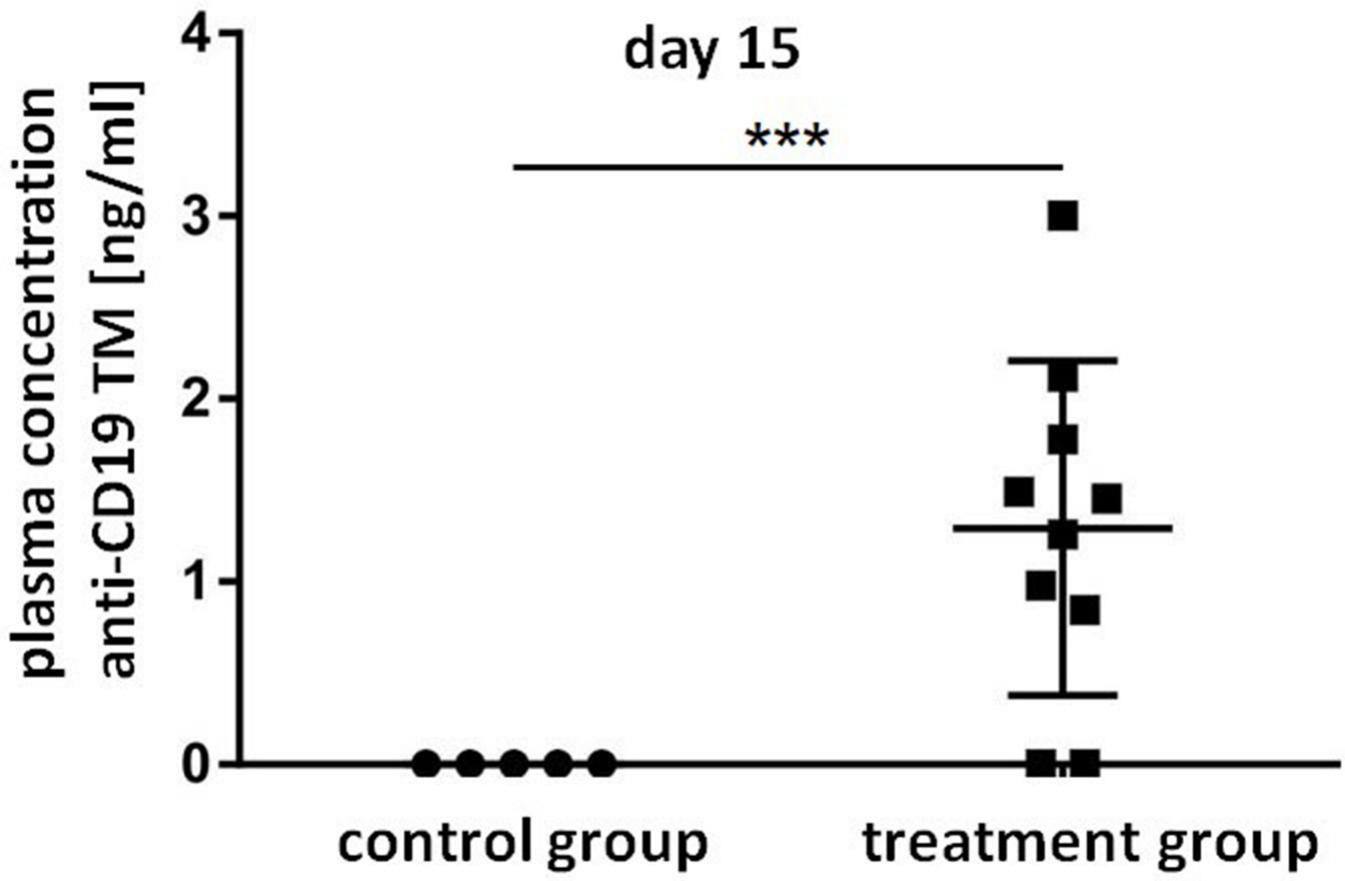
Plasma concentrations of the anti-CD19 TM released from cryogel-housed producer cells Plasma concentration of the anti-CD19 TM in the control and the treatment group at the endpoint of the experiment (15 days post-transplantation) was quantified via ELISA. Data are shown as mean ± SD of five or ten experimental mice, respectively. Statistical significance between control group and treatment group was determined by unpaired, two-tailed Student’s *t*-test (^***^p < 0.001).

As mentioned above, we recently established a cryogel system allowing us to house cells for *in vivo* synthesis of a bsAb at a concentration sufficient for retargeting of T cells to tumor cells in experimental mice [42, 43]. For proof of concept, in the current manuscript we wanted to learn whether or not a similar approach may also work for an *in vivo* synthesis of TMs for a prolonged attack of tumor cells by UniCAR T cells. Moreover, we wanted to know whether we can use the TM-producing cell line for this purpose at least in an experimental animal approach. In first preliminary experiments we estimated the amount of TM that can be released from 3T3 cells housed in a cryogel (data not shown). Based on these data, we calculated that around 5 × 10^5^ 3T3 cells might be able to produce and release an amount of the anti-CD19 TM necessary for retargeting of UniCAR T cells in experimental mice (data not shown). Following the scheme already shown in Figure 1C, ten NMRI^nu/nu^ mice were s.c. transplanted with starPEG-heparin cryogels housing 5x10^5^ anti-CD19 TM-producing 3T3 cells (Figure 6, treated animals). Five NMRI^nu/nu^ mice s.c. inoculated with empty scaffolds served as controls (Figure 6, control animals). Six days after implantation, both animal groups were s.c. injected into their right legs with a mixture of 1x10^6^ UniCAR CD28/ζ-engineered T cells and 1 × 10^6^ CD19 positive Nalm-6 cells expressing firefly luciferase. Luminescence imaging of anesthetized mice was performed 8h and 24h after transfer of tumor and T cells. For this purpose, 200 μl of D-luciferin potassium salt were i.p. injected ten minutes before aquiring luminescence signals [39] [see also MATERIALS AND METHODS]. The optical imaging data of control and treated animals illustrated in Figure 6 show an efficient killing of CD19 positive tumor cells in the treated mice group (*n* = 10) but not in the control group (*n* = 5). In order to further confirm this TM-dependent killing we estimated the plasma concentration of the anti-CD19 TM in the control and treatment group at the endpoint of the experiment (15 days post-transplantation) by ELISA (Figure 7). As expected, anti-CD19 TMs were only detected in plasma samples of treated animals. Furthermore, in the peripheral blood of eight out of ten mice which were implanted with the starPEG-heparin cryogel housing the 3T3 producer cells, we estimated a concentration of the anti-CD19 TM that was between EC_50_ and EC_100_ or even higher (Figure 7).

## DISCUSSION

Clinical studies using CAR T cells directed against CD19 have demonstrated the impressive therapeutic potential of CAR-modified T cells in patients [23–27]. Stable clinical remissions of B-lineage leukemias could be achieved even in patients after all standard therapies had failed. Unfortunately, severe side effects can occur soon after adoptive T cell transfer but also after an efficient therapy with CAR T cells. Adverse reactions that emerge after tumor cell eradication e.g. caused by cross-reactivy of the CAR with healthy tissues may be avoided by elimination of engineered T cells. Severe or even life-threatening side effects that occur shortly after the adoptive transfer of conventional CAR T cells are usually caused by the massive release of inflammatory cytokines or due to lysis of large amounts of tumor cells. For obvious reasons, it is hard to predict the initial response of conventional CAR T cells with respect to expansion, cytokine secretion and tumor cell killing. One way to reduce these early risks would be to allow for adoptively transferred CAR T cell activity to be switched on and off at will. Such a repeatable stop and go therapy, however, cannot be realized with conventional CAR T cells. In order to (i) avoid a complete elimination of the genetically modified CAR T cells and (ii) realize a rapid shut off of CAR T cells, in 2014 we presented a switchable modular CAR platform termed UniCAR [35]. The UniCAR system originated from a modular BiTE format [36, 37, 39]. As already summarized in the introduction section, UniCAR-equipped T cells are not directed to a cell surface target antigen and, therefore, are per se inert. They become active only in the presence of a TM and will automatically be turned off after its elimination. So the steering of UniCARs depends on the elimination of the TM. The problem going along with the feature of a rapid elimination is that the TM has to be given to the patient as a permanent infusion in order to reach sufficiently high concentrations in the tumor. Since presentation of the UniCAR system, related CAR platforms have been described [46, 47]. In 2016 so called sCARs were introduced [46]. Like UniCARs sCARs recognize a peptide epitope. Moreover, sCAR-TMs are fusion molecules of a peptide epitope and an Ab domain that is, in contrast to UniCAR-TMs, related to Y-shaped full-size Abs. As full-size Abs have a long elimination time of up to several weeks, the application of such sCAR-TMs may be more convenient for patients than UniCAR-TMs. However, their precise control especially during early treatment phase of patients might be more critical and, therefore, such systems should behave more like conventional CAR T cells. Most suitable for patients would be a switchable CAR system behaving like the UniCARs during early treatment phase and like sCARs once most of the tumor cells have been destroyed. Especially in case of relatively save target antigens such as CD19 a combination of short- and long-lasting TMs could be of interest. One way to accomplish this goal for the UniCAR system would be to extend the elimination time e.g. by PEGylation of UniCAR-TMs or by construction of Y-shaped UniCAR-TMs related to conventional Abs. Here we describe a continuous *in vivo* synthesis of a low-molecular TM as an alternative strategy. For this purpose, we implanted experimental mice with a cryogel in which genetically modified producer cells were housed. These cells were able to release sufficient amounts of the TM protein for retargeting of UniCAR T cells to tumor cells. Regarding a first-line treatment with the rapidly eliminated TMs, the risk of cytokine release storm and tumor lysis syndrome should be reduced. However, after eradication of most of the tumor cells, the implantation of such a TM-releasing scaffold could circumvent the application of the TM via continous infusion without putting the patient at high risk for severe side effects. UniCAR T cells could still be rapidly shut down in case unwanted long-term immune responses against healthy tissues occur simply by removal of the scaffold. Furthermore, the here presented *in vivo* factory may also be helpful to improve currently available but limited animal models for modular CAR treatments such as the UniCAR system. For animal care reasons it is not possible to repeatedly inject or infuse the TMs in experimental mice. Using a cell-seeded scaffold producing the TM *in vivo* could probably allow the development of improved treatment models.

In summary, we here describe a novel scFv-based TM directed to CD19 for retargeting of UniCAR T cells to CD19 positive leukemic cells. In agreement with previous studies, the most relevant cytokines released by UniCAR T cells stimulated via the anti-CD19 TM were GM-CSF, IL-2, TNF and IFN-γ. Release of IL-6 was not detected. Moreover, the same cell line used for *in vitro* production of the TM could also be housed in a cryogel and implanted into experimental mice for sustained *in vivo* synthesis of the TM.

## MATERIALS AND METHODS

### Ethics statement

Human peripheral blood mononuclear cells (PBMCs) were isolated from buffy coats supplied by the German Red Cross (Dresden, Germany). The study, including the consent form, was approved by the local ethics committee of the university hospital of the medical faculty of Carl-Gustav-Carus TU-Dresden (EK27022006).

### Cell lines

The human acute lymphoblastic leukemia (ALL) cell line Nalm-6 (ATCC CRL-3273) was cultured in complete RPMI 1640 medium supplemented with 10% FCS, 1% non-essential amino acids, 1 mM sodium pyruvate, 2 mM N-acetyl-_L_-alanyl-_L_-glutamine 100 µg/ml penicillin and 100 μg/ml streptomycin (all purchased from Biochrom AG, Berlin, Germany). For the bioluminescence-based *in vivo* assays Nalm-6 cells were transduced with the gene encoding firefly luciferase (Nalm-6 luc). Transduction was performed using a lentiviral packaging system as described previously (see also below) [45, 48]. Human Embryonic Kidney cells HEK293T (ATCC CRL-11268) and murine 3T3 cells (ATCC CRL-1658) were cultured in DMEM medium (ThermoFisher Scientific, Schwerte, Germany) supplemented with 10% FCS, 100 µg/ml penicillin/streptomycin and 1% non-essential amino acids.

All cells were maintained at 37°C in a humidified atmosphere of 5% CO_2_.

### Generation of recombinant anti-CD19 TM-releasing mouse 3T3 cells

For *in vitro* and *in vivo* expression of the anti-CD19 TM, the reading frame encoding the recombinant Ab construct was cloned into the self-inactivating lentiviral vector p6NST50. Lentiviral particles pseudotyped with the Vesicular Stomatitis Virus envelope (VSV-G) were generated by transient transfection of HEK293T cells [48]. Virus supernatant was harvested and used for transduction of murine 3T3 cells as described previously [48].

### Expression, purification and analysis of the anti-CD19 TM

The His-tagged anti-CD19 TM released by gene-modified 3T3 cells was purified from culture supernatants using single-step affinity chromatography on Ni-NTA columns (Qiagen, Hilden, Germany). Isolated TMs were analyzed by Western blotting as previously described [8, 9]. The amount of secreted anti-CD19 TM was quantified by enzyme-linked immunosorbent assay (ELISA) as described previously [42, 43]. Standard samples were prepared as a 2-fold serial dilution of the purified anti-CD19 TM starting from 100 ng/ml.

### Fabrication of macroporous starPEG-heparin cryogel scaffolds and seeding of producer cell line

Macroporous starPEG-heparin cryogels were prepared and producer cells were seeded as described in detail recently [43].

Briefly, network formation via chemical crosslinking (carbodiimide chemistry) of 4-arm amino terminated starPEG (molecular mass 10,000 g/mol; JenKem Technology, USA) and heparin (molecular mass 14,000 g/mol; Merck, Germany) was combined with cryogelation technology [43]. The resulting dry cryogel cylinders were cut in slices of 1 mm thickness and punched into 3 mm diameter discs (scaffolds). After washing and swelling in phosphate buffered saline (PBS, pH 7.4), the cryogels were sterilized with ethanol (70%) overnight. To improve cell adhesion, the starPEG-heparin cryogels were biofunctionalized with a fibronectin-derived peptide sequence (H_2_N-GWGGRGDSP-CONH_2_, molecular mass 886.92 g/mol, synthesized in house, using 300 µl of a 80 µg/ml solution) as previously described [43].

5 × 10^5^ 3T3 cells were seeded per cryogel following the procedure reported previously [42].

### Isolation of T cells and lentiviral transduction

Isolation of human CD3^+^ T cells occurred from freshly prepared human PBMCs of healthy, consenting volunteers using the Pan T cell isolation kit, human (Miltenyi Biotec, Bergisch Gladbach, Germany). Prior to transduction, isolated T cells were cultured in RPMI 1640 complete medium supplemented with 200 U/ml IL-2 (Proleukin^®^ S, Novartis Pharma GmbH, Nuremberg, Germany), 5 ng/ml IL-7 and 5 ng/ml IL-15 (ImmunoTools, Friesoythe, Germany) at densities of 1x10^6^ cells/ml. Production of lentiviral particles and transduction of primary human T cells was carried out as described before [48].

### Flow cytometry analysis

Isolated T cells were stained with fluorochrome-labeled mAbs directed against human CD4/FITC (clone VIT4), CD3/Vioblue (clone BW264/56) and CD8/APC (clone BW135/80, all purchased from Miltenyi Biotec). For analysis of T cell activation, cells were stained with anti CD3/Pacific Blue mAb (clone UCHT1, BD Bioscience), CD4/PE-Cy7 mAb (clone SK3, BD Bioscience) and CD25/PE mAb (clone BC96, BD Bioscience). For analysis of CD19 expression, Nalm-6 cells were stained with anti-CD19/PE mAb (clone LT19, Miltenyi Biotec). In order to assess binding of the anti-CD19 TM, Nalm-6 cells were incubated with 20 µg/ml of the recombinant protein, followed by the mAb La5B9 directed against the UniCAR epitope (E5B9) [49] and a PE-conjugated anti-mouse IgG secondary Ab (Beckmann Coulter, Krefeld, Germany). Samples were analyzed using a MACSQuant^®^ Analyzer and MACSQuantify^®^ software (Miltenyi Biotec) [49]. For estimation of UniCAR cell surface expression, the hinge region of the UniCAR contains the peptide tag E7B6 which, like the UniCAR epitope (E5B9), is taken from the nuclear antigen La/SS-B [48, 50].

### Flow cytometry-based cytotoxicity assay

The killing of CD19 positive tumor cells by redirected T cells was measured using a previously described flow cytometry-based assay [36, 38]. For this purpose, target cells were labeled with eFluor670^®^ (eBioscience, ThermoFisher Scientific) prior to incubation with anti-CD19 TM and/or effector T cells in an assay volume of 200 µl. After 24h and 48h of coculture the surviving tumor cells were estimated using a MACSQuant^**®**^ Analyzer and shown as living cells/µl of assay.

Patient-derived tumor cells and autologous T cells were obtained by FACS technology as described previously [36]. For this purpose cells were stained with fluorescently labeled anti-CD3, anti-CD19 and anti-CD45 Abs. CD45^+^CD3^+^ T cells served as autologous effector cells. CD45^+^CD3^-^ MLL cells or CD45^+^CD19^+^CD3^-^ ALL cells were defined as target cells, respectively.

### Cytokine release assay

UniCAR T cells were cultivated with or without target cells at an e:t ratio of 5:1 either in the presence or absence of 30 nM anti-CD19 TM. After 24h, cell-free supernatants were harvested and analyzed using the MACSPlex Cytokine 12 Kit, human, a MACSQuant^®^ Analyzer and the MACSQuantify^®^ software (all from Miltenyi Biotec) according to the manufacturer‘s instructions [39, 40].

### Optical imaging of tumor xenografted mice

All animal experiments were carried out at the Helmholtz-Zentrum Dresden-Rossendorf (HZDR) according to the guidelines of German Regulations for Animal Welfare and have been approved by the Landesdirektion Dresden (24-9165.40-4, 24.9168.21-4/2004-1). Four weeks old female NMRI-Foxn1^nu^/Foxn1^nu^ mice were purchased from JANVIER LABS (St. Berthevin, France). General anesthesia was induced with 10% (v/v) and maintained with inhalation of 8% (v/v) desflurane (Suprane, Baxter, Germany) in 30/10% (v/v) oxygen/air. Luminescence imaging of the subcutaneous injected cells (exposure times 1 s, 10 s, and 60 s) was performed using a dedicated small animal multimodal imaging system (*In-Vivo*-Xtreme, Bruker, Germany) 10 min after i.p. injection of 200 µl of D-luciferin (15 mg/ml) (ThermoFisher Scientific). In parallel, an X-ray photograph was taken from the same animals at the same position [39–41].

### Statistical analysis

Statistical analysis was performed using GraphPad Prism 7 software (GraphPad Software Inc., La Jolla, CA, USA). Statistical tests were applied as indicated in Figure legends. *P* values < 0.05 were considered significant.

## SUPPLEMENTARY MATERIALS FIGURE


