# Supplementary material for: Retargeting of UniCAR T cells with an in vivo synthesized target module directed against CD19 positive tumor cells
Source: Oncotarget. 2017 Dec 21;9(7):7487–500. doi: 10.18632/oncotarget.23556 (PMC5800918; doi:10.18632/oncotarget.23556)
Supplement: Supplementary file 1 [file oncotarget-09-7487-s001.pdf]

Retargeting of UniCAR T cells with an *in vivo* synthesized target module directed against CD19 positive tumor cells

SUPPLEMENTARY MATERIALS

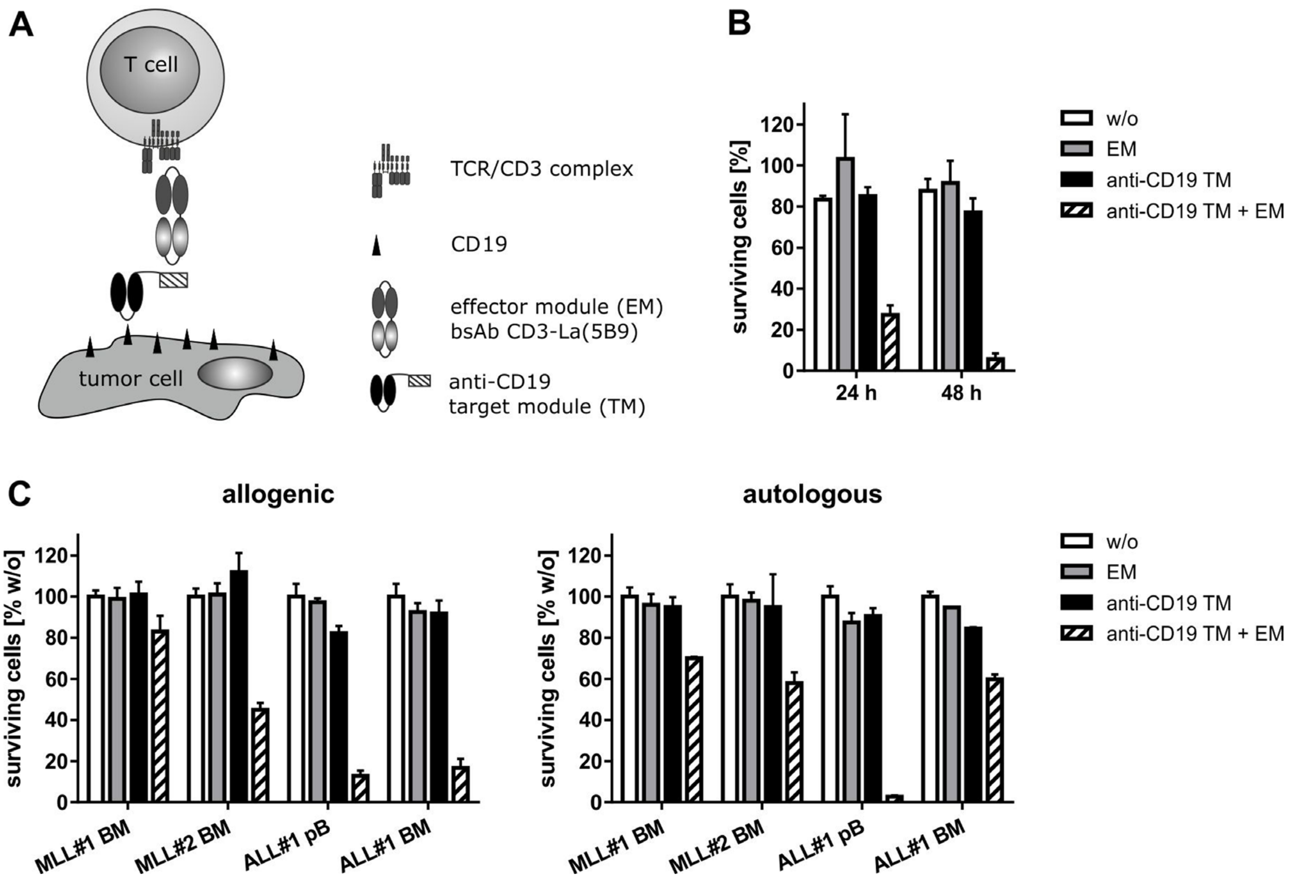

Supplementary Figure 1: Efficient lysis of CD19 positive tumor cells by using the anti-CD19 TM in combination with our modular BiTE system. (**A**) The anti-CD19 TM can also be used in combination with a universal EM representing a conventional bsAb that simultaneously binds to the UniCAR-tag of the TM and to CD3 of T cells.36,37,40 The resulting Ab complex can cross-link T cells and CD19 positive tumor cells resulting in efficient tumor cell lysis. (**B**) Tumor cell killing was analyzed by co-incubation of human pan T cells and fluorescently labeled CD19 positive Nalm-6 tumor cells in the presence or absence of 30 pmol/ml Ab at an effector-to-target cell ratio of 5:1. As negative control Nalm-6 cells were additionally cultured without T cells and Ab (Data not shown). Corresponding to this control assay the relative percentage of surviving tumor cells was calculated for all samples. Data show results of triplets for one donor. (**C**) Tumor cells were isolated from bone marrow (BM) or peripheral blood of patients with MLL or ALL by using FACS technology. In a flow cytometry-based killing assay fluorescently labeled, patient-derived CD45+CD3- MLL or CD45+CD3-CD19+ cells were incubated with equal amounts of either human pan T cells from healthy donors (left panel, allogenic) or with sorted, autologous CD45+CD3+ T cells (right panel, autologous) in the presence or absence of 30 pmol/ml Ab for 40 h. Number of cells of the control assay (w/o) performed in the absence of Ab was set to 100%. Relative to this control percentage of surviving cells for all samples was calculated. Results of three different donors are shown.
